# The impact of non-significant variable decelerations appearing in the latent phase on delivery mode: a prospective cohort study

**DOI:** 10.1186/1477-7827-8-81

**Published:** 2010-07-05

**Authors:** Raed Salim, Gali Garmi, Zohar Nachum, Eliezer Shalev

**Affiliations:** 1 Department of Obstetrics and Gynecology, HaEmek Medical Center, Afula, Israel and Rappaport Faculty of Medicine, Technion, Haifa, Israel

## Abstract

**Background:**

Variable decelerations are the most frequent fetal heart rate changes that are related to labor. The objective of the study was to estimate the impact of non-significant variable decelerations (NSV) appearing during the latent phase of labor on delivery mode and neonatal outcome.

**Methods:**

Women at term, who were in the latent phase of labor and had a singleton pregnancy, were prospectively included. Women were divided into three groups. All had a fetal heart rate tracing with normal baseline and variability. The study group was composed of women who had in addition NSV, Category II, according to the National Institute of Child Health and Human Development categorization system. Women who had Category I tracings composed the control group. Women who had non-repetitive severe variables (SV) composed a second control group (Category II-SV). Main outcome compared was mode of delivery. Secondary outcome was cord pH. One-way analysis of variance was used to compare the continuous demographic and clinical variables of the three groups. Backwards stepwise logistic regression using significant univariables was performed to determine which predicted operative delivery. P < 0.05 was considered significant.

**Results:**

Of 1005 women who delivered during the study period 186 had Category II- NSV tracings (study group), 76 had Category II-SV and 251 had Category I tracings. Mode of delivery and indications for operative delivery were similar between women in Category II-NSV compared to Category I. In addition mean cord pH did not differ between the two groups. Conversely, women in Category II-SV, had a higher rate of cesarean or vacuum deliveries compared to the other groups (p = 0.0001). Beside, they had a significantly higher number of neonates born with cord pH between 7.0 to 7.1 (p = 0.03).

**Conclusions:**

Non-significant variable decelerations in early stages of labor are probably a non-ominous sign for neonatal outcome and have no impact on delivery mode.

## Background

Variable decelerations are defined as an abrupt (onset to nadir of less than 30 sec) decrease of 15 beats per minute or more of the FHR below the baseline, and lasting 15 seconds or more but less than two minutes in duration [1]. Variable decelerations are the most frequent fetal heart rate (FHR) changes that are related to labor [2]. The management and impact of variable decelerations on neonatal outcome are equivocal. Early investigators agreed that fetal depression was more likely to be associated with severe variable decelerations, which they confirmed by measuring fetal scalp pH [3].

In 2008, the National Institute of Child Health and Human Development (NICHD) Workshop Report on FHR monitoring proposed a "three-tier FHR interpretation system" [1]. Variable decelerations, regardless of their type, in the presence of normal variability were categorized under Category II. However, no evidence of the utility or predictive value and therefore the management of these FHR tracings were presented.

In this study we aimed to investigate whether a difference exists in the progression of FHR tracing to the point that required operative delivery among women who had non-significant variable decelerations early in the latent phase of labor compared to women who had a reassuring FHR tracing. In addition we aimed to explore whether these FHR abnormalities have any impact on mode of delivery and neonatal outcome.

## Methods

A prospective study was held from January to April 2009 in the labor and delivery ward of the department of Obstetrics and Gynecology at Ha'Emek Medical Center in Afula, Israel, a university teaching hospital. Women at term (gestational age of 37 weeks or more) who were in the latent phase of labor and had a singleton pregnancy were included. The latent phase was defined as the interval between the start of regular contractions (women's report) combined with any cervical dynamics (dilatation and/or effacement) until the active phase of labor was established when cervical dilatation was greater than 4 cm. Variable decelerations were defined according to the 2008 National Institute of Child Health and Human Development workshop report on electronic fetal monitoring [1]. Variable decelerations were categorized [4] as significant (SV) if the FHR reached 70 beats per minute for one minute or more but less than two minutes, otherwise, they were categorized as non-significant (NSV).

Women, who had NSV, episodic or recurrent, in addition to normal baseline and moderate variability, categorized among Category II tracings according to the 2008 NICHD three-tier system" [1], composed the study group (Category II- NSV). Women who had Category I tracings composed the control group (Category I). Women with SV which appeared at a frequency of one in 10 minutes or less and who had also a moderate variability and normal baseline in FHR tracing, categorized among Category II tracings according to the 2008 NICHD three-tier system" [1], composed a second control group (Category II-SV).

Women were excluded if they had other FHR tracing abnormalities during the latent phase. In addition, women who had a cesarean section without a trial of labor, delivered infants with major malformations were also excluded. All women had a continuous FHR tracing during the first and second stages of labor running at a speed of 1 cm per minute (Philips, Series 50 IP-2; Hewlett Packard, Boeblingen, Germany).

All tracings were assessed by two expert obstetricians at the same time, who were both masked as to the trial groups and neonatal outcomes.

Main outcome compared between the groups was mode of delivery. Secondary outcomes studied included Apgar score and cord pH. Other parameters collected and compared between the groups were maternal age, parity, body mass index (BMI), maternal disease (hypertensive disorders, gestational or pre-gestational diabetes, thrombophilia, cardiac disease and asthma), the presence of: oligohydramnios defined as amniotic fluid index (AFI) equal or less than five cm, polyhydramnios defined as AFI greater than 23 cm (determined before admitting to the delivery ward) and induction of labor.

Criteria for arrest that require operative delivery were in accordance with the American College of Obstetrics and Gynecology guidelines [5]. Women who developed late or recurrent severe variable decelerations during the first stage of labor that did not resolve with additional intrauterine resuscitative therapies (i.e. encouraging women to adopt the left lateral position, treatment of maternal hypotension, administrating facial oxygen and lowering or discontinuing of labor stimulation) were delivered abdominally. Similar tracing were allowed to continue in the second stage of labor if the variability and baseline FHR were normal; otherwise operative delivery (vacuum or cesarean delivery) was conducted. Operative delivery was also employed in both stages of labor in cases where Category III tracings appeared and did not resolve with additional intrauterine resuscitative therapies. Only vacuum extraction was used in cases of operative vaginal delivery and it was performed when fetal position was two cm lower than the level of the ischial spines. Fetal blood sampling was not available during the study period.

### Institutional Review Board

The local Institutional Review Board approved the study.

### Statistical analysis

One-way analysis of variance was used to compare the continuous demographic and clinical variables of the three groups. Significant group differences were then tested (post-hoc) by Bonferonni multiple comparisons. Categorical variables were analyzed by chi-square tests or Fisher's exact test where warranted. Backwards stepwise logistic regression using significant univariables was performed to determine which predicted operative delivery. This was repeated for cesarean section vs. vaginal delivery.

Independent sample t-tests were used to test for differences in the continuous demographic and clinical variables between those with pH < 7.1 and pH > 7.1 as well as between neonates with low (< 2500 g) and normal birth weight. Backwards stepwise logistic regression using significant variables was performed to determine which variables predicted pH < 7.1 and which predicted low birth weight. Significance was defined as p value < 0.05.

We estimated an incidence of 5% for operative delivery among women who had Category I tracings. In order to demonstrate a difference of 10% in the rate of operative deliveries between Category I and Category II-NSV tracings (the study group), i.e., a 15% rate of operative deliveries, with an alpha of 0.05 and a power of 80% a sample size of 160 per group was required.

## Results

Of the 1005 women delivered during the study period, 513 were eligible for the study. Of the 492 women who were excluded, 263 were admitted in the active phase of labor, 161 were delivered by cesarean section without a trial of labor, 34 were delivered preterm, 25 were in the latent phase but had other FHR tracing abnormalities, 6 were multiple gestations and 3 women had a termination of pregnancy. Of the 513 women who were included, 186 had NSV (Category II-NSV), 76 had SV (Category II-SV) and 251 had no decelerations (Category I). Of all the 513 women included, 444 (86.5%) had spontaneous vaginal delivery, 25 (4.9%) delivered by vacuum and 44 (8.6%) delivered abdominally. All women who had a vacuum attempt delivered vaginally.

Comparisons of demographic and clinical data between the three groups are presented in table 1. The incidence of cord pH between 7.0 and 7.1 was greater in the Category II-SV group than either of the other two groups (p = 0.03). None of the neonates were born with cord artery pH less than 7.0 and none had an Apgar score of less than 7 at 5 minutes. Stepwise logistic regression using the three groups and delivery method as potential predictors revealed that delivery method (i.e. vacuum) was the only significant predictor (p = 0.0001).

**Table 1:** Demographic and clinical data associated with fetal heart rate category

	Category I	Category II	Category II	**p**^**a**^
		NSV	SV	
	(N = 251)	(N = 186)	(N = 76)	
**Maternal characteristics**				
Maternal age, years	29.7 ± 5.9	29.6 ± 5.6	29.6 ± 5.2	0.9
Body mass index, kg/m^2^	29.3 ± 4.5	29.2 ± 4.8	30.8 ± 5.3	0.05^b^
Gestational age, weeks	39.6 ± 1.2	39.4 ± 1.5	39.6 ± 1.3	0.5
Parity	2.6 ± 1.6	2.5 ± 1.5	2.3 ± 1.5	0.4
Primiparous vs Multiparous				0.05^b^
Primiparous	72 (28.7)	56 (30.1)	33 (43.4)	
Multiparous	179 (71.3)	130 (69.9)	43 (56.6)	
Maternal disease	20 (8.0)	39 (21.0)	14 (18.4)	0.0001^c^
Oligohydramnios	13 (5.2)	21 (11.7)	18 (23.7)	0.0001^d^
Polyhydramnios	6 (2.4)	4 (2.2)	2 (2.6)	1
Induction of labor	62 (24.7)	121 (65.1)	58 (76.3)	0.0001^c^
Epidural	61 (24.3)	61 (32.8)	32 (42.1)	0.0001^c^
**Delivery method**				0.0001^b^
Spontaneous Vaginal	238 (94.8)	166 (89.2)	40 (52.6)	
Vacuum or cesarean	13 (5.2)	20 (10.8)	36 (47.4)	
Vacuum	6 (2.4)	8 (4.3)	11 (14.5)	
Cesarean	7 (2.8)	12 (6.5)	25 (32.9)	
Reasons for vacuum or cesarean delivery				0.03^b^
Non reassuring fetal heart rate monitoring	3 (23.1)	5 (25.0)	20 (55.6)	
Failure to progress in the active or second stage	10 (76.9)	15 (75.0)	16 (44.4)	
**Neonatal outcome**				
Neonatal weight, g	3329 ± 392	3297 ± 439	3130 ± 487	0.002^b^
Neonates born < 2500 g	2 (0.8)	7 (3.8)	9 (11.8)	0.0001^e^
Apgar score at 5 minutes	9.96 ± 0.23	9.90 ± 0.31	9.86 ± 0.39	0.01
Cord pH	7.31 ± 0.07	7.31 ± 0.07	7.30 ± 0.08	0.5
Cord pH between 7.0 and 7.1	2 (0.8)	1 (0.5%)	4 (5.3)	0.008^b^
Meconium stained amniotic fluid	22 (8.8)	26 (14.0)	15 (19.7)	0.03^f^
Nuchal cord or true knot	23 (9.2)	19 (10.2)	12 (15.8)	0.3
Neonatal deaths	0	0	0	1

Data are mean ± standard deviation or n (%) unless otherwise specified.

^a^Comparison of all three groups; ^b^Category II-SV versus Category I and Category II-NSV; ^c^Category II-NSV and Category II-SV versus Category I; ^d^Category II-NSV versus Category I, p = 0.01, ^d^Category II-SV versus Category II-NSV, p = 0.02;

^e^Category II-NSV versus Category I, p = 0.04, ^e^Category II-SV versus Category II-NSV, p = 0.02; ^f^Category II-SV versus Category I.

Abbreviations: NSV: non-significant variable decelerations (study group); SV: significant variable decelerations; NS: non-significant. Maternal disease: hypertensive disorders, gestational or pre-gestational diabetes, thrombophilia, cardiac disease and asthma.

### Operative delivery

Reasons for an operative delivery (table 1) were non reassuring fetal heart rate monitoring according to our protocol or failure to progress in the active or second stages. Reasons for an operative delivery differed between the 3 groups (p = 0.03). Post-hoc testing revealed that the difference between Category I and Category II-NSV groups was not significant. Table 2 presents the number and rate of cesarean and/or vacuum delivery by group. Next, backwards stepwise logistic regression was performed using BMI, parity, maternal diseases, polyhydramnios, oligohydramnios, and the three groups as potential risk factors in order to predict an operative delivery. Induction and epidural were forced into the model in order to account for differences in the incidence of induction between the 3 groups. Holding all other variables equal, the odds of an operative delivery for a woman experiencing SV tracings was nearly 14 times (p = 0.0001; OR: 13.6; 95% CI: 5.7-32.5) that of a woman with no decelerations (Category I) and 8 times that of a women with NSV tracings (p = 0.0001; OR: 8.0; 95% CI: 3.74-20.0). Whereas a significant difference was observed previously in the rate of operative delivery between the Category I and Category II-NSV groups (table 2), this difference became not significant (p = 0.2) after adjustment for epidural use, induction, BMI, maternal disease, polyhydramnios, and primiparous/multiparous delivery. In addition, the odds of having an operative delivery was nearly 1.5 times greater than having a spontaneous vaginal delivery for an increase of 5 kg/m ^2 ^in BMI (p = 0.02; OR: 1.5; 95%; CI: 1.1-2.0). The odds of an operative delivery were 6.7 times higher in primiparous over multiparous deliveries (p = 0.0001; OR: 6.7; 95%; CI: 3.3-13.6). The odds of having an operative delivery were 4.5 times higher among women who had at least one maternal disease (p = 0.0001; OR: 4.5; 95%; CI: 2.0-9.8). In addition, the odds of having an operative delivery were more than 5 times higher among women who had polyhydramnios (p = 0.03; OR: 5.4; 95%; CI: 1.2-24.9).

**Table 2:** Number and prevalence of cesarean and vacuum delivery by group

	Number (%)	OR	95% CI	p
**Cesarean or vacuum**				0.0001
Category I	13 (5.2%)	1.00	Reference	
Category II-NSV	20 (10.8%)	2.21	1.07-4.56	0.03
Category II-SV	36 (47.4%)	16.48	8.04-33.76	0.001
**Cesarean**				0.0001
Category I	7 (2.8%)	1.00	---------	
Category II-NSV	12 (6.5%)	2.25	0.80-6.87	0.1
Category II-SV	25 (32.9%)	17.09	6.65-48.78	0.0001
**Vacuum**				
Category I	6 (2.4%)	1.00	---------	
Category II-NSV	8 (4.3%)	1.84	0.55-6.53	0.3
Category II-SV	11 (14.5%)	6.91	2.23-23.47	0.0001

Abbreviations: OR: odds ratio; CI: confidence interval; NSV: non-significant variable decelerations; SV: significant variable decelerations.

### Cesarean delivery

Backwards stepwise logistic regression was performed using BMI, gestational week, parity, maternal disease, polyhydramnios and oligohydramnios, as well as the three groups as potential risk factors in order to predict cesarean delivery. Epidural use and induction were forced in the model. Holding all other variables equal, the odds of a cesarean delivery for a woman experiencing SV tracings was nearly 13 times (p = 0.0001; OR: 12.7; 95% CI: 4.3-37.5) that of a woman with no decelerations (Category I) and 7.8 times that of a women with NSV tracings (p = 0.0001; OR: 7.8; 95% CI: 3.1-20). There was no significant difference between Category I and Category II-NSV groups (p = 0.5). In addition, the odds of having a cesarean delivery was 1.8 times greater than having a spontaneous vaginal delivery for an increase of 5 kg/m ^2 ^in BMI (p = 0.001; OR: 1.8; 95%; CI: 1.3-2.6). The odds of a cesarean delivery were 4.1 times higher in primiparous over multiparous deliveries (p = 0.001; OR: 4.1; 95%; CI: 1.7-9.7). The odds of a cesarean delivery increase with advance gestational age (p = 0.04; OR: 1.4; 95%; CI: 1.02-2.0). The odds of having a cesarean delivery were 6.3 times higher among women who had at least one maternal disease (p = 0.0001; OR: 6.3; 95%; CI: 2.5-16.2). In addition, the odds of having a cesarean delivery were more than 9 times higher among women who had polyhydromnia (p = 0.01; OR: 9.6; 95%; CI: 1.7-52.7) and 2.6 times higher among women who had oligohydramnios (p = 0.05; OR: 2.6; 95%; CI: 1.01-6.9).

Using the significant predictors that were associated with operative delivery and cesarean delivery, a multivariable logistic regression equation was built to predict the probability of delivering operatively or by cesarean section. Examples of the equation's predictive capability are shown in table 3 and 4 by calculation of the probability of operative or cesarean delivery for four hypothetical pregnant women. The full logistic regression equation is also shown.

**Table 3:** Probability* of having an operative delivery for four hypothetical women with different combinations of predictors

Hypothetical	Ep	In	BMI	Primi	MD	Polyhy	Group	p
Woman 1	yes	no	23	no	no	no	NSV	6.7%
Woman 2	yes	no	23	no	no	no	SV	37.6%
Woman 3	no	yes	35	yes	yes	yes	NSV	98.5%
Woman 4	no	yes	35	yes	yes	yes	SV	99.8%

Abbreviations: P: probability (%); e: exponential function (exp); Ep: 1 if woman has an epidural and 0 otherwise; In: 1 if the women is induced and 0 otherwise; BMI: woman's body mass index in kg/m^2^; Primi: 1 if woman is primiparous and 0 otherwise; MD: 1 if women has one or more of the following diseases: diabetes, hypertensive disorders, thrombophilia, cardiac disease and asthma, and 0 otherwise; Polyh: 1 if woman has polyhydramnios and 0 if not; NSV: 1 if the woman has non-significant variable decelerations and 0 otherwise; SV: 1 if the woman has significant variable decelerations and 0 otherwise.

**Table 4:** Probability* of having a cesarean section for four hypothetical women with different combinations of predictors

Hypothetical	Ep	In	BMI	GA	Primi	MD	Polyhy	Oligoh	Group	p
Woman 1	no	no	25	40	no	no	no	no	SV	8%
Woman 2	no	no	25	40	no	no	no	no	NSV	1%
Woman 3	yes	yes	35	40	yes	yes	no	yes	SV	89.2%
Woman 4	yes	yes	35	40	yes	yes	no	yes	NSV	49.3%

Abbreviations: P: probability(%); e: exponential function (exp); Ep:1 if woman has an epidural and 0 otherwise; In: 1 if the women is induced and 0 otherwise; BMI: woman's body mass index in kg/m2; GA: gestational age in weeks; Primi: 1 if woman is primiparous and 0 otherwise; MD: 1 if women has one or more of the following diseases: diabetes, hypertensive disorders, thrombophilia, cardiac disease and asthma, and 0 otherwise; Polyhy: 1 if woman has polyhydramnios and 0 if not; Oligoh: 1 if woman has olighydramnios and 0 if not; NSV: 1 if the woman has non-significant variable decelerations and 0 otherwise; SV: 1 if the woman has significant variable decelerations and 0 otherwise.

## Discussion

Variable decelerations are a common finding during the intrapartum FHR monitoring. It has been reported that more than 40% of intrapartum FHR tracings had some degree of variable decelerations pattern [2]. In the current study 51% of women admitted to the delivery ward in the latent phase of labor had any variable decelerations in FHR tracing. The management and impact of variable decelerations on neonatal outcome are equivocal and different studies reported inconsistent results [2,6,7].

The NICHD categorized all types of variable decelerations among Category II, in the presence of normal variability, because there is not adequate evidence to classify these as Category I or Category III [1]. In the present study we showed that women with NSV tracings, episodic or recurrent, appearing in early stages of labor had a favorable neonatal outcome and had a comparable incidence of operative delivery compared to women who had a reassuring FHR tracings (Category I). Moreover, the percentage of FHR tracings that worsened during the course of labor and that led to an operative delivery was also similar between the two groups.

Conversely, women with SV tracings had, compared to the other two groups, a higher rate of operative deliveries although most women with SV in the present study delivered vaginally healthy neonates. Vacuum delivery was the most significant factor that predisposed to a higher incidence of more acidotic neonates probably due to delayed delivery as compared to an earlier delivery by cesarean section. The incidence of operative delivery increased significantly when a combination of distinct maternal characteristics and obstetric conditions, were met.

It has been reported that during the course of normal labor, there are intermittent reductions of placental gas exchange as a direct consequence of uterine contractions and consequent reductions in uterine or fetal placental blood flow and fetal oxygenation. This reduction even in normal uncomplicated labor is associated with a consistent fall in pH and a rise in base deficit. However, if the fetus is healthy, with a normal placental reserve, it may be able to stably adapt to deep brief decelerations for prolonged periods [8-10]. In this study we included women in the latent phase only to investigate the impact of non-significant variable decelerations appearing at early stages on the course of labor. We showed that the presence of non-significant variable decelerations with normal FHR base line and variability at early stages of labor did not adversely affect the course of labor compared to Category I tracing.

Several studies have linked between maternal obesity and delivery by cesarean section, mostly due to prolonged labor and failure to progress [11,12]. In addition to the association with operative deliveries, obesity was correlated in this study, with a higher incidence for the presence of SV. While it has been reported that labors that failed to progress during the first stage of labor were more associated with non-reassuring FHR patterns [5], and that a higher incidence of stillbirth was observed among obese compared with normal weight women [13], further studies are needed to investigate the exact correlation between obesity and the presence of SV.

In this study we investigated only one aspect, however frequent, of Category II tracings, and the results of this study and the model produced to predict the probability for operative delivery may assist the clinician to provide proper counseling to woman encountering this type of FHR abnormality during early stages of labor. The advantage of this model that was produced is that it includes factors that are all available at early stages of labor, allowing caregivers the possibility of providing accurate counseling early during the labor course regarding mode of delivery. Indeed, this model can provide physicians and patients alike with a reasonably accurate assessment of a woman's chance of delivering operatively.

Nevertheless, this model cannot be used to predict outcome for all women i.e. women who are preterm or have other FHR tracing abnormalities other than variable decelerations at early stages of labor. Future analyses will need to ascertain if its results are equally valid among patient populations in different hospital settings.

## Conclusions

Non-significant variable decelerations in early stages of labor are probably a non-ominous sign for neonatal outcome and have no significant impact on mode of delivery compared to normal FHR tracings. We propose categorizing the NSV tracings into Category I, leaving only tracings with SV in Category II.

## Competing interests

The authors declare that they have no competing interests.

## Authors' contributions

RS conceived and designed the study, supervised the data collection, assisted in the analysis and drafted the manuscript. ZN and GG participated in the design of the study, assisted in collection and maintenance of the data and co-wrote the article. ES assisted in conceiving, designing and analysis, and edited the manuscript. All authors read and approved the final manuscript.

## Authors' information

RS is a lecturer, Rappaport Faculty of Medicine Technion, Israel Institute of Technology, senior obstetrician, head of delivery ward, Department of Obstetrics and Gynecology Ha'Emek Medical Center, Afula, Israel; GG is a resident, Department of Obstetrics and Gynecology Ha'Emek Medical Center, Afula, Israel; ZN is a lecturer, Rappaport Faculty of Medicine Technion, Israel Institute of Technology, senior consultant obstetrician, Department of Obstetrics and Gynecology Ha'Emek Medical Center, Afula, Israel and ES is a professor and associate Dean, Rappaport Faculty of Medicine Technion, Israel Institute of Technology, Chairman, Department of Obstetrics and Gynecology Ha'Emek Medical Center, Afula, Israel.
